# Transcriptomic Investigation of the Virus Spectrum Carried by Midges in Border Areas of Yunnan Province

**DOI:** 10.3390/v16050674

**Published:** 2024-04-25

**Authors:** Lifen Yang, Weichen Wu, Sa Cai, Jing Wang, Guopeng Kuang, Weihong Yang, Juan Wang, Xi Han, Hong Pan, Mang Shi, Yun Feng

**Affiliations:** 1 Yunnan Provincial Key Laboratory for Zoonosis Control and Prevention, Yunnan Institute of Endemic Disease Control and Prevention, Dali 671000, China; yanglifen2989@163.com (L.Y.); cais5209@163.com (S.C.); kgp0317@163.com (G.K.); yangwh0604@163.com (W.Y.); wj2502@163.com (J.W.); hanxi922@163.com (X.H.); panhong6662022@163.com (H.P.); 2 State Key Laboratory for Biocontrol, School of Medicine, Shenzhen Campus of Sun Yat-sen University, Sun Yat-sen University, Shenzhen 518107, China; wuweixiongde@126.com (W.W.); wangj796@mail2.sysu.edu.cn (J.W.); 3 School of Public Health, Dali University, Dali 671000, China; 4 State Key Laboratory of Remote Sensing Science, Center for Global Change and Public Health, Faculty of Geographical Science, Beijing Normal University, Beijing 100875, China

**Keywords:** transcriptome, midges, viruses, arboviruses, Yunnan

## Abstract

Yunnan province in China shares its borders with three neighboring countries: Myanmar, Vietnam, and Laos. The region is characterized by a diverse climate and is known to be a suitable habitat for various arthropods, including midges which are notorious for transmitting diseases which pose significant health burdens affecting both human and animal health. A total of 431,100 midges were collected from 15 different locations in the border region of Yunnan province from 2015 to 2020. These midges were divided into 37 groups according to the collection year and sampling site. These 37 groups of midges were then homogenized to extract nucleic acid. Metatranscriptomics were used to analyze their viromes. Based on the obtained cytochrome C oxidase I gene (COI) sequences, three genera were identified, including one species of *Forcipomyia*, one species of *Dasyhelea,* and twenty-five species of *Culicoides*. We identified a total of 3199 viruses in five orders and 12 families, including 1305 single-stranded positive-stranded RNA viruses (+ssRNA) in two orders and seven families, 175 single-stranded negative-stranded RNA viruses (−ssRNA) in two orders and one family, and 1719 double-stranded RNA viruses in five families. Six arboviruses of economic importance were identified, namely Banna virus (BAV), Japanese encephalitis virus (JEV), Akabane virus (AKV), Bluetongue virus (BTV), Tibetan circovirus (TIBOV), and Epizootic hemorrhagic disease virus (EHDV), all of which are capable, to varying extents, of causing disease in humans and/or animals. The survey sites in this study basically covered the current distribution area of midges in Yunnan province, which helps to predict the geographic expansion of midge species. The complexity and diversity of the viral spectrum carried by midges identified in the study calls for more in-depth research, which can be utilized to monitor arthropod vectors and to predict the emergence and spread of zoonoses and animal epidemics, which is of great significance for the control of vector-borne diseases.

## 1. Introduction

The midges (*Ceratopogonidae*) are medically important insects with piercing–sucking mouthparts, widely distributed worldwide, and diverse in species. They not only cause direct nuisance and irritation to humans and animals through their biting but can also transmit various pathogens through blood-sucking, leading to associated diseases. Midges are significant vector insects closely related to human and animal diseases [1,2]. Research on *Ceratopogonidae* in China began in 1958, achieving significant advancements in fields such as taxonomy, ecology, and genetics in recent years. Currently, more than 6000 species of midges have been identified [3]. They can be divided into two major categories: blood-sucking midges and non-blood-sucking midges. The blood-sucking midges include the genera *Culicoides*, *Lasiohelea*, *Leptoconops,* and *Austroconops*, of which there are 1719 known species worldwide [4]. China has reported 443 species of blood-feeding midges, mainly belonging to the genera *Forcipomyia*, *Leptoconops*, and *Culicoides* [5]. These blood-sucking midges are important vector insects that can bite and feed on blood from both humans and animals, posing a threat to their well-being. Global warming has led to the expansion of the distribution range and activity season of midges, resulting in an increase in the variety of diseases transmitted by them [6]. At present, five virus families and nine genera have been identified from blood-sucking midge samples, including the families *Reoviridae*, *Bunyaviridae*, *Flaviviridae*, and *Togaviridae* [7,8,9,10,11]. These viruses mainly infect livestock such as cattle, sheep, and horses, while some of them can also infect humans. In China, viruses from the family *Reoviridae* [11,12], *Togaviridae* [8], and *Flaviviridae* [13] have been isolated. Furthermore, bacteria and parasites, including *Lactobacillus* [14], *Protozoa* [15], and *Nematodes* [16] have been identified. Despite the identification of multiple pathogens, the possibility of undiscovered pathogens still exists.

In recent years, with the acceleration of global warming, the abundance and distribution of midges have dramatically increased, and researchers have continued to intensify their studies of midges, with the concomitant revelation of new midge species and the pathogens they carry [17]. Although progress has been made in the research of *Ceratopogonidae* and disease associations in China, the risk of introducing non-endemic midge-borne diseases has increased with the growth of international trade. Therefore, we need to strengthen immigration and quarantine measures and conduct in-depth investigations on the distribution, species, and carried pathogens of midges to understand the patterns and provide a theoretical basis for controlling midge-borne infectious diseases. Yunnan province has a complex and diverse climate, harboring a wide variety of blood-sucking midge species and various midge-borne viruses, including Bluetongue virus, Banna virus, Tibetan orbivirus, Japanese encephalitis virus, and Flock House virus, among others [18,19,20,21]. Yunnan province shares borders with Guizhou, Guangxi, Sichuan, Tibet, Myanmar, Laos, and Vietnam, and it has 25 bordering counties with Myanmar, Laos, and Vietnam, spanning 4060 km. Consequently, we conducted investigations in specific border regions of Yunnan province to comprehend the distribution patterns of midge-borne viruses, improve our understanding of midge-borne infectious diseases, prevent their transmission, and reduce their impact on public health and the economy.

## 2. Materials and Methods

### 2.1. Sample Collection and Processing

**Figure 1:**
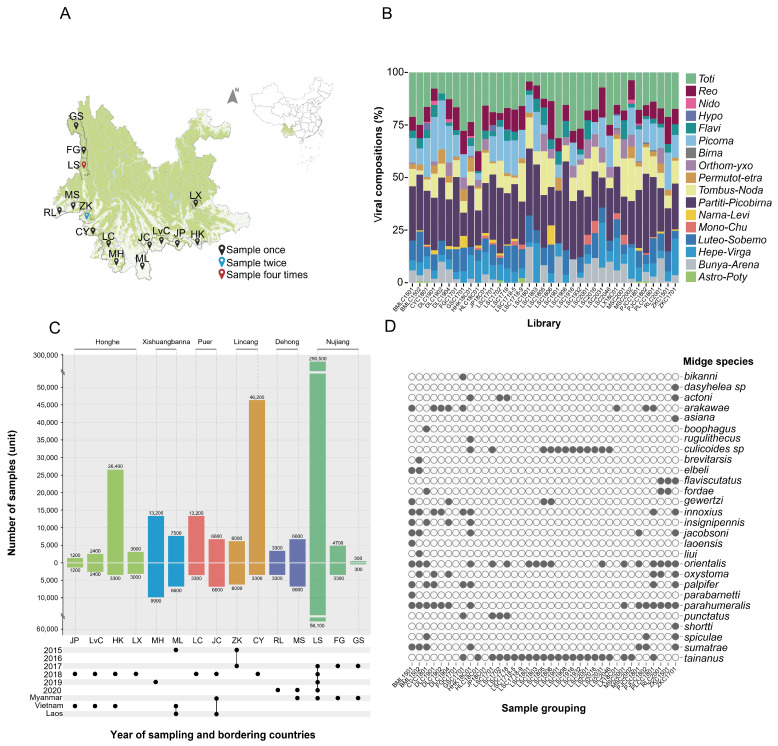
Overview of the samples and 37 libraries analyzed in this study. (**A**) In locations where midges were, light trap collections were made. From southeast to northwest, Jinping (JP), Lvchun (LvC), Hekou (HK), and Luxi (LX) in Honghe Prefecture; Menghai (MH) and Mengla (ML) in Xishuangbanna; Lancang (LC) and Jiangcheng (JC) in Pu’er City; Zhenkang (ZK) and Cangyuan (CY) in Lincang; Ruili (RL) and Mangshi (MS) in Dehong Prefecture and Lushui (LS), Fugong (FG) and Gongshan (GS) in Nujiang Prefecture. Different sampling frequencies are represented by colors: black once, blue twice, and red four times. (**B**) Virus composition. The composition of viruses in the 37 libraries was determined based on the relative proportions of reads corresponding to each viral branch hit by overlapping clusters. The x axis is the names of the 37 libraries constructed. The legend on the right side provides abbreviations for the 5 virus orders and 12 virus families obtained in this study: Astro-Potyviridae (Astro-Poty), Birnaviridae (Birna), Bunya-Arenavirales (Bunya-Arena), Flaviviridae (Flavi), Hepe-Virgaviridaea (Hepe-Virg), Hypoviridae (Hypo), Luteo-Sobemoviridae (Luteo-Sobem), Mono-Chuvirales (Mono-Chu), Narna-Leviviridae (Narna-Levi), Nidovirales (Nido), Orthomyxoviridae (Orthomyxo), Partiti-Picobirnaviridae (Partiti-Picobna), Permutotetraviridae (Permutotetra), Picornavirales (Picorna), Reovirales (Reo), Tombus-Nodaviridae (Tombus-Noda), and Totiviridae (Toti). (**C**) The number of midges collected at 15 sampling points and the number of midges used for library construction. The number of midges collected in this study is shown above the *x*-axis, and the number of midges used for library construction is shown below the *x*-axis. Bars with the same color represent sampling sites belonging to the same prefecture, as labeled above the bars. The sampling years and neighboring countries are annotated below the chart using black dots. (**D**) Classification and identification information of midges. Each column represents a library, the library name is marked at the end of each column, and each row represents a midge. Each gray dot indicates that the corresponding midge exists in the corresponding library.

From 2015 to 2020, we collected samples from a total of 15 sampling points in the border areas of Yunnan province, China (Figure 1A). Ultraviolet mosquito lamps(12 V, 300 mA, Model: LTS-M02; Wuhan Lucky Star Environmental Protection Tech Co., Wuhan, China) were used near cattle and pig farms to collect insect. No collection medium was used during the collection process, which lasted continuously from 5:00 PM to 9:00 AM the following day for two consecutive nights after freezing at −20 °C. The midges were identified to genus level using morphological characteristics, transported to the laboratory in liquid nitrogen, and stored in refrigerators at −80 °C to preserve their integrity. The quantity of libraries to be established in each area was determined based on the environmental conditions, sampling frequency, and sample size. Each library consists of 3300 midges. For areas with a sample size exceeding 3300 midges, libraries are built through random sampling. All samples are included in the library for areas where the sample size is less than 3300 midges.

### 2.2. Virus Nucleic Acid Extraction and Gene Library Construction

Virus RNA was extracted from the ground supernatant of midges using the RNeasy Plus Universal Mini Kit from Qiagen, Germany. The gene library was constructed using the Zymo-Seq RiboFree™ Total RNA library Kit from Zymo Research, Irvin, CA, USA.

### 2.3. Virus Sequencing and Analysis

Sequence assembly of the obtained sequencing data involved several steps and bioinformatics tools to ensure the accuracy and reliability of the results. First, raw sequencing reads were pre-processed using Trimmomatic to remove adapter sequences and low-quality reads [22]. The high-quality reads were then de novo assembled into contigs using Trinity [23]. Next, the assembled contigs were subjected to sequence alignment against the NCBI non-redundant nucleotide database (nt), non-redundant protein database (nr), and conserved domain database (CDD) using BLASTn, BLASTx, and other methods. Only the contigs with high credibility (e-value < 1 × 10^−5^) and alignment to known viral sequences were retained [24]. These retained viral sequences were extracted and aligned using the SeqMan program from the DNAStar software package to obtain complete nucleotide sequences for various viral segments.

Bowtie2 was used to remove reads mapped to the host cell ribosomal RNA in the library, and then the remaining reads were mapped again to the obtained viral sequences. The number of reads mapped to the reference sequences was counted, and the assembled consensus sequences were used as the final viral sequences [25]. The sequencing depth and average coverage of each viral segment in the library were calculated using Geneious Prime software. The coding regions and predicted proteins for each segment were identified using the ORF finder tool and Protein BLAST feature on the NCBI website. Further analysis was performed using various bioinformatics software, including Xshell7, Xftp7, Perl, Mafft, and DNAstar [26]. Nucleotide sequences of contigs longer than 1000 base pairs were extracted and translated into amino acid sequences after removing redundancy. The conserved domains of each sequence were identified by comparing them with the Conserved Domain Database (CDD). Additionally, a systematic phylogenetic tree was constructed by aligning the sequences with protein reference sequences downloaded from NCBI.

## 3. Results

### 3.1. Sample Collection and Identification

From 2015 to 2020, we conducted extensive midge collection efforts, totaling 431,100 midges, at 15 sampling points in six border prefectures and cities within Yunnan province (Figure 1A,C). In Lushui County of Nujiang State, we conducted continuous collection efforts over four years (2017–2020), amassing the greatest quantity of midge samples, totaling an impressive 290,500 individuals, with an annual average of 72,625 individuals collected each year. In Zhenkang County of Lincang City, we conducted two sampling events (once in 2015 and once in 2017), collecting 3000 midges each year, totaling 6000 over the two years. At the remaining sampling sites, we conducted one sampling event each. Specifically, in Menglai in 2015, we collected 7500 midges; in Fugong and Gongshan in 2017, we collected 4700 and 300 midges, respectively, with Gongshan having the lowest midge collection in this study. In 2018, our midge collections were diverse: in Jinping, Lvchun, Hekou, Luxi, Lancang, Jiangcheng, and Cangyuan, we gathered 1200, 2400, 26,400, 3000, 113,200, 6600, and 46,200 midges, respectively. Subsequently, in 2019, we collected 13,200 midges in Menghai, followed by 3300 and 6600 midges in Ruili and Mangshi, respectively, in 2020. 

According to the library construction principle, this study included a total of 115,200 midges for library construction, resulting in 37 libraries (Figure 1C). Jinping included 1200 midges to construct one library (JP18C01), Lvchun included 2400 midges to construct one library (HLC18C01), Hekou included 3300 midges to construct one library (HHK18C01), and Luxi included 3000 midges to construct one library (LX18C01). Menghai included 9900 midges to construct three libraries (DLC1901, DLC1902, DLC1904), Mengla included 6600 midges to construct two libraries (BMLC1501, BMLC1502), and Lancang included 3300 midges to construct one library (PLCC1801). Jiangcheng included 6600 midges to construct two libraries (PJCC1801, PJCC1802), Zhenkang included 6000 midges to construct two libraries (ZKC1501, ZKC1701, with 3000 midges in each library), and Cangyuan included 3300 midges to construct one library (CYC1801). Ruili included 3300 midges to construct one library (RLC2001), Mangshi included 6600 midges to construct two libraries (MSC2001, MSC2002), and Lushui included 56,100 midges to construct 17 libraries (LSC1701, LSC1702, LSC1718, LSC1718-5, LSC1718-9, LSC1801, LSC1803, LSC1805, LSC1806, LSC1901, LSC1908, LSC1916, LSC1932, LSC2001, LSC2016, LSC2031, LSC2046). Fugong included 3300 midges to construct one library (FGC1701), and Gongshan included 300 midges to construct one library (GSC1701). 

To gain insights into the midge species present in the collected samples, we used partial samples for library construction, which led to the successful identification of 28 different species of midges (Figure 1D), classified into three genera: *Forcipomyia* (1), *Dasyhelea* (1), and *Culicoides* (26). *Culicoides* adults are crepuscular and/or nighttime feeders, whereas *Forcipomyia* adults tend to be more active during the daytime [3]. Notably, the highest species diversity was found in Mengla County, Xishuangbanna Prefecture where we identified 15 species. In contrast, the species diversity of midges collected in Lvchun County and Luxi County of Honghe Prefecture was found to be the lowest, and only one species of midge was identified in each location. The remaining 12 sampling sites, except for Jinping and Fugong, were all successfully identified. Among them, Menghai (6), Hekou (9), Gongshan (6), Lushui (4), Jiangcheng (6), Lancang (7), Mangshi (3), Ruili (5), Cangyuan (8), and Zhenkang (14). The three most frequently identified species in the libraries were *Culicoides tainanus*, *Culicoides parahumeralis,* and *Culicoides orientalis*. *Culicoides tainanus* was identified in 22 libraries, *Culicoides parahumeralis* was found in 14 libraries, and *Culicoides orientalis* was present in 16 libraries. Conversely, *Forcipomyia bikanni*, *Dasyhelea sp*, *Culicoides asiana*, *Culicoides boophagus*, *Culicoides rugulithecus*, *Culicoides brevitarsis*, *Culicoides elbeli*, *Culicoides laoensis*, *Culicoides liui*, *Culicoides parabarnetti*, and *Culicoides shortti* were the lowest observed midges, each appearing in only one library.

### 3.2. Viruses Carried by Midges

Paired-end (150 bp) sequencing of each RNA library was performed using the Illumina Novaseq 6000 platform, resulting in a total of 1,361,136 pathogenic contigs. Among these contigs, 243,543 were viral contigs, accounting for 17.9% of the total contigs. To construct a systematic evolutionary tree, we selected contigs with a length greater than 1000 base pairs, totaling 15,996 contigs for analysis. 

In this study, a total of 3199 RNA viruses were obtained from the samples, belonging to 5 viral orders and 12 viral families, including *Astro-Potyviridae* (Astro-Poty), *Birnaviridae* (Birna), *Bunya-Arenavirales* (Bunya-Arena), *Flaviviridae* (Flavi), *Hepe-Virgaviridaea* (Hepe-Virg), *Hypoviridae* (Hypo), *Luteo-Sobemoviridae* (Luteo-Sobem), *Mono-Chuvirales* (Mono-Chu), *Narna-Leviviridae* (Narna-Levi), *Nidovirales* (Nido), *Orthomyxoviridae* (Orthomyxo), *Partiti-Picobirnaviridae* (Partiti-Picobna), *Permutotetraviridae* (Permutotetra), *Picornavirales* (Picorna), *Reovirales* (Reo), *Tombus-Nodaviridae* (Tombus-Noda), and *Totiviridae* (Toti). The most abundant virus species belonged to the Partiti-Picobirna clade (*n* = 837), followed by Toti (*n* = 532), while Birna had the lowest representation (*n* = 5). Astro-Poty and Nido both had six species. Additionally, two orders and seven families had less than 100 virus species, including Birna, Astro-Poty, Nido, Mono-Chu, Narna-Levi, Permutotetra, Orthomyxo, Flavi, and Hypo (Figure 1B).

**Figure 2:**
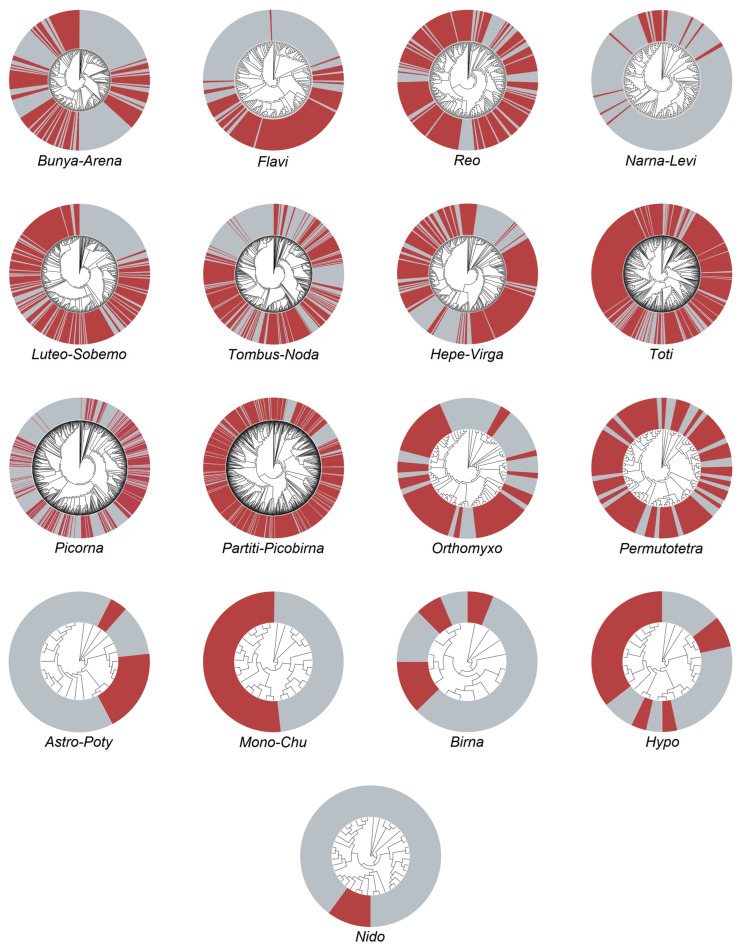
RNA viruses exhibit diverse phylogenetic diversity. In each tree, red represents the virus sequence information obtained in this study, and gray represents the reference sequence information. The names of each viral order or family are abbreviated and placed below the phylogenetic tree. Astro-Potyviridae (Astro-Poty), Birnaviridae (Birna), Bunya-Arenavirales (Bunya-Arena), Flaviviri-dae (Flavi), Hepe-Virgaviridaea (Hepe-Virg), Hypoviridae (Hypo), Luteo-Sobemoviridae (Luteo-Sobem), Mono-Chuvirales (Mono-Chu), Narna-Leviviridae (Narna-Levi), Nidovirales (Nido), Or-thomyxoviridae (Orthomyxo), Partiti-Picobirnaviridae (Partiti-Picobna), Permutotetraviri-dae (Permutotetra), Picornavirales (Picorna), Reovirales (Reo), Tombus-Nodaviridae (Tombus-Noda), and Totiviridae (Toti).

The 37 gene libraries resulted in a total data size of 185.81 G compressed. The pathogenic contigs amounted to 1,361,136, with a total of 2,304,534,936 reads. Among these, the viral contigs accounted for 243,543, which represents 17.9% of the total contigs. The RNA viruses obtained were classified into three types: 1305 species of single-stranded positive-sense RNA viruses (+ssRNA) from two viral orders and seven viral families, 175 species of single-stranded negative-sense RNA viruses (−ssRNA) from two viral orders and one viral family, and 1719 species of double-stranded RNA viruses (dsRNA) from one viral order and four viral families (Figure 2).

### 3.3. Phylogenetic Analysis of Vector-Borne Viruses

**Figure 3:**
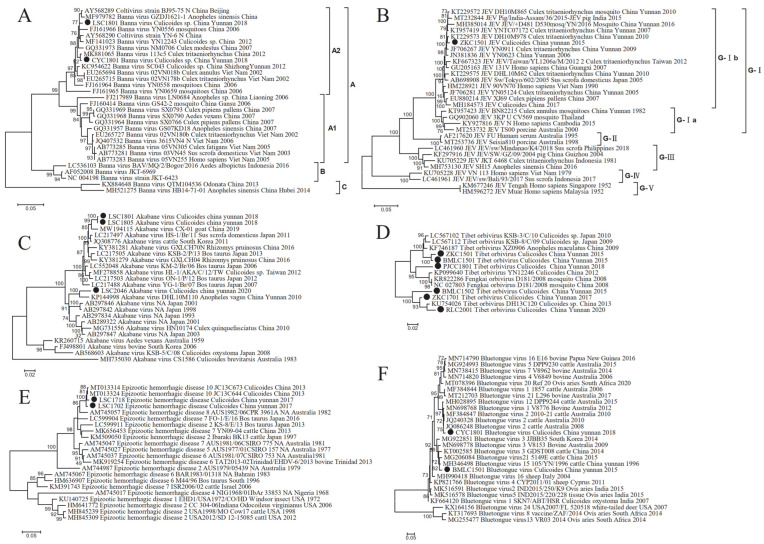
Analysis of insect-borne virus system evolution. (**A**) Phylogenetic tree of Seg-12 of two BAV strains. (**B**) Phylogenetic tree of nucleotide sequence of a JEV strain in Zhenkang County. (**C**) Phylogenetic tree of nucleotide sequence of AKV M fragment of three strains. (**D**) Phylogenetic tree of nucleotide sequence of Seg-1 of six strains of TIBOV. (**E**) Phylogenetic tree of nucleotide sequence of the Seg-1 of three strains of EHDV. (**F**) Phylogenetic tree of nucleotide sequence of Seg-8 of two BTV strains.

In our study, we conducted extensive midge collection efforts in 15 border areas of Yunnan province and identified six vector-borne viruses: Banna virus (BAV), Japanese encephalitis virus (JEV), Akabane virus (AKV), Tibet orbivirus (TIBOV), Epizootic hemorrhagic disease virus (EHDV), and Bluetongue virus (BTV). BAV, JEV, AKV, and EHDV were detected exclusively in areas bordering Myanmar. The BAV sequences obtained from 12 gene segments revealed three major evolutionary branches: A, B, and C genotypic branches. The A2 genetic subtype was closely related to virus strains from mosquitoes. In this phylogenetic tree, all BAVs form three major evolutionary branches: A, B, and C genotypic branches. The virus sequences obtained in this study belong to the A2 genetic subtype and are most closely related to virus strains obtained from mosquitoes (Figure 3A). We also obtained the JEV nucleotide sequence from the ZKC1501 sample library, which has a length of 10,961 bp. Using this virus sequence and nucleotide sequences of 29 JEV reference strains from GenBank, we constructed another phylogenetic tree. This tree shows that the ZKC1501 strain belongs to the same evolutionary branch as the G-Ireference strains, and is classified into the G-I genotype. The G-I genotype is further divided into two subtypes: G-Ia and G-Ib [27,28]. This strain belongs to the G-Ib subtype and is distantly related to JEV strains of genotypes II, III, IV, and V. However, this strain is closely related to the JEV genotype I-b isolates of strain JF706267 (YN0911) from San-dai-beak mosquitos in Mile County, Yunnan province in 2009, and strain KT229573 (DH10M978) from San-dai-beak mosquitos in Ruili City, Dehong Prefecture, in 2010 (Figure 3B). In our study, we obtained three strains of M-segment virus from the libraries LSC1801, LSC1805, and LSC1806. Among them, both LSC1801 and LSC1805 libraries obtained sequences of S-segment and M-segment. Through evolutionary analysis of the obtained M-segment, we found that the sequences from the LSC1801 and LSC1805 libraries are closely related to the M-segment of the 2019 goat virus strain MW194115 (CX-01). However, the relationship of LSC2046 to LSC1801 and LSC1805 is distant, while it is most closely related to virus strains obtained from 2010 Yunnan Dehong wandering mosquitoes (Figure 3C). Finally, we obtained EHDV Seg-1, Seg-2, Seg-3, and Seg-4 virus sequences from the gene libraries of Lushui and Daluo. We constructed a phylogenetic tree for the Seg-1 sequence with the reference sequences. From the tree, it can be seen that these two virus strains are closely related and have a close relationship. Among them, the strains LSC1718, LSC1702, MT013324 (JC13C644), and MT013314 (JC13C673) from mosquitoes in Yunnan in 2013 are most closely related (Figure 3E). These findings provide us with a deeper understanding and help us better study and control these viruses.

The remaining two viruses, TIBOV and BTV, were detected in areas bordering Vietnam, Myanmar, and Laos. Additionally, all the obtained TIBOV sequences in this study belong to the Seg-1 virus. By constructing a phylogenetic tree with reference strains from GenBank, we found the following relationships: ZKC1501 and BMLC1501 are closely related to the Japanese *Culicoides* isolate LC567102(KSB-3/C/10); PJCC1801 shows a close relationship with the Chinese *Culicoides* isolate KP099640(YN12246); BMLC1502, ZKC1701, and RLC2001 are most closely related to the 2013 *Culicoides* isolate KU754026(DH13C120) (Figure 3D). The Bluetongue virus obtained the Seg-8 viral sequence and constructed a phylogenetic tree. The tree showed that CYC1801 is closely related to Australian viral strains, particularly with the 2008 bovine strain JQ086248. BMLC1501 is closely related to Chinese viral strains, especially with the 1996 bovine strain MH346498 (105/YN/1996) and the 2015 bovine strain MG206084 (5149E) isolated in Yunnan (Figure 3F).

## 4. Discussion

### 4.1. Diversity of Midge Species

There are four genera of blood-sucking midges globally: *Culicoides*, *Lasiohelea*, *Leptoconops*, and *Austroconops*. Among these, *Culicoides* stands out as the largest, most widely distributed, and diverse genus, with the closest association to humans and livestock [29]. Molecular classification research on midges began with the examination of genetic differences in *Culicoides variipennis* in 1992 [30]. Recent technological advancements have enabled the utilization of COⅠ and COⅡ genes of mitochondrial DNA and internal transcribed spacer of ribosomal DNA for midge taxonomy [31]. Since the proposal of DNA barcoding technology and its global research [31,32], the COⅠ gene, serving as a standard target gene, has spurred the development of molecular biology-based species identification techniques. This technology has now been extensively studied and applied in classifying and identifying vertebrates and insects, such as mosquitoes [30]. Pages N successfully employed COⅠ gene barcode technology for the molecular biological classification and identification of midges, suggesting its efficacy as a genetic marker for inter-species identification of blood-sucking midges [33]. However, to date, there have been no reports on the use of COⅠ gene barcode technology for midge classification and identification in China.

In this study, we obtained COⅠ gene sequences of midges and identified 28 species belonging to three genera: *Forcipomyia* (1), *Dasyhelea* (1), and *Culicoides* (26). *Forcipomyia* and *Dasyhelea* primarily feed on plant sap, while *Culicoides* are blood-sucking midges. However, we encountered challenges in identifying the species of midges collected in Jinping County and Fugong County through COⅠ gene sequencing, possibly due to the specific gene type used for species identification [31]. Despite this challenge, our data provide valuable information about the diversity and distribution of midge species in the border regions of Yunnan province, contributing significantly to our understanding of midge populations and their potential implications for public health and the environment. The most diverse midge collections were collected in Mangla County, Xishuangbanna Prefecture, in 2015, where we identified 15 species. Our findings contrasted with those of Liu et al. in the same year [34], revealing several species that they did not collect, such as *Culicoides brevitarsis*, *Culicoides gewertzi*, *Culicoides innoxius*, *Culicoides laoensis*, and *Culicoides sumatrae*. These differences can be attributed to varying sampling habitats suggesting abundant midge species in Mangla County [35]. Notably, *Culicoides laosensis*, mainly distributed in Fujian, China, Laos, and Indonesia, was observed in Yunnan for the first time, possibly due to the unique geographical location of southeastern Mangla County, bordering Laos [36]. A continuous 4-year midge sample collection in Lushui revealed previously uncollected and newly discovered midge species through molecular biology identification. However, the species diversity of midges was the lowest in Lvchun County and Luxi County of Honghe Prefecture. Only one species of midge was identified in each location, *C.taiananus* from Lvchun County and *C.arakawae* from Luxi County, respectively. Two years of collection in Zhenkang County yielded an increasing number of midge species over time. *Culicoides orientalis*, *Culicoides parahumeralis*, *Culicoides palpifer*, *Culicoides arakawae*, and *Culicoides tainanus* were dominant species with a wide distribution. A more comprehensive understanding of midge species distribution in Yunnan province’s border areas requires further analysis and research. Additionally, long-term monitoring of midge distribution and viral status in neighboring areas is necessary to mitigate the occurrence of infectious diseases caused by midges.

Sample collection was conducted twice in Zhenkang (once in 2015 and once in 2017), with approximately 3000 midges collected each year. Three species of midges were identified from samples collected in 2015, while 13 species of midges were identified from samples collected in 2017, with 12 of them being blood-sucking midges. The *Culicoides fordae* collected in 2015 was not found in 2017. Over a year apart, the number of midge species in the same location continues to increase, reaching more than 10 species. This indicates an increase in midge species in the area. In order to prevent potential hazards, further research on the distribution and viral carriage of midges in this region and neighboring areas is needed. Among the collected midge samples, *Culicoides orientalis*, *Culicoides parahumeralis*, *Culicoides palpifer*, *Culicoides arakawae*, and *Culicoides tainanus* were dominant species with a wide distribution. To gain a more comprehensive understanding of the distribution of midge species in the border areas of Yunnan province, further analysis and research are required. Additionally, long-term monitoring of midge distribution and viral status in bordering areas is necessary to reduce the occurrence of infectious diseases caused by midges.

### 4.2. Viral Diversity

We revealed the metatranscriptomic profile of RNA viruses carried by midges in the border areas of Yunnan, China. Transcriptome analysis of the collected samples revealed the presence of multiple RNA viruses, classified according to the Baltimore classification into positive-sense single-stranded RNA viruses, negative-sense single-stranded RNA viruses, and double-stranded RNA viruses. According to the ICTV classification, this study obtained 5 viral orders and 12 viral families. The most abundant clade, *Partiti-Picobirnaviridae*, consisted of 837 virus species and has a host range including plants, fungi, protists, insects, and vertebrates. Insects, as hosts of these viruses, have a mutualistic relationship with them. These viruses were first detected in *Drosophila melanogaster* in 2015 using metagenomics and deep sequencing techniques [37]. In the same study, viruses belonging to families *Luteo-Sobemoviridae*, *Flaviviridae*, *Picornaviridae*, *Permutotetraviridae*, *Bunyavirales*, *Birnaviridae*, *Reoviridae*, and *Totiviridae* were also detected [37,38,39], and these viruses were identified in our current research as well. With the wide application of genomics and gene sequencing technologies, more scholars have discovered many previously unknown or known but not extensively studied viruses. In this study, we employed genomics, deep sequencing, and other methods to conduct a systematic research analysis on the collected samples. We detected up to 800 species of *Partiti-Picobirna* clade viruses in the collected midges, indicating their survival in insects other than fruit flies. However, the specific relationship between the viruses and their hosts, as well as their role in diseases, requires further in-depth research. The second-largest group of viruses after the *Partiti-Picobirna* clade in terms of quantity is the Toti family, which was also detected in fruit flies in our study. This suggests that Toti family viruses can survive in fruit flies and other midges, potentially beyond the previously discovered fungi and protozoa [40].

We also identified 35 species of Hypo and *Narna-Levi* family viruses in the collected samples. Further exploration is needed to determine whether these viruses are carried by the midges themselves or by fungi and bacteria on the midges, and whether they can be transmitted to other hosts through certain pathways when parasitic on midges. Additionally, we identified six known common arboviruses from the Bunya-Arena order, Reo order, and *Flavi* family. The viruses under the Bunya-Arena order have infectivity and pathogenicity to humans and animals, suggesting potential risks of infection and pathogenicity [41]. In this study, we obtained 116 virus species within the order, including common arboviruses such as Akabane virus. A total of 341 virus species were identified in the Reo order and *Flavi* family, including common arboviruses such as JEV, BAV, EHDV, TIBOV, and BTV. Among the 3199 virus species obtained, only six common arboviruses were identified and analyzed, while detailed species identification was not performed for other viruses. Further research is needed to understand the characteristics, host range, and potential pathogenicity of these unidentified viruses.

### 4.3. Diversity of Arboviruses

In our study, we collected samples and identified sequences from different arbovirus genera through deep sequencing. These included BAV, EHDV, TIBOV, BTV, JEV, and AKV. The presence and distribution of these viruses exhibit significant geographical features. For example, BAV, JEV, AKV, and EHDV were detected only in areas bordering Myanmar. Specifically, in Cangyuan County and Lushui County, the BAV sequences we obtained belong to the A2 genotype and have a close genetic relationship with southern strains. There is high homology with Yunnan strains, indicating the regional distribution of BAV. Notably, these two counties belong to different climatic types, and BAV has been detected in various climate conditions such as tropical, subtropical, and temperate regions in China, suggesting its strong adaptability to diverse climates and environments. Furthermore, we found that the Beijing strain AY568289 in China can cluster with southern strains in genotype A2. Both genotype A1 and A2 clusters include strains from Vietnam, indicating no clear habitat barrier between these strains (Figure 3A). In this study, we obtained 12 segment gene sequences of BAV from midges collected in Cangyuan County and Lushui City, Yunnan province. This further confirms that midges can carry BAV and may transmit diseases. Therefore, strengthening monitoring and control of midges and their BAV infection in this region is necessary. Additionally, we also noted that JEV is widely distributed in China, especially in Yunnan province, a high-risk area [42,43]. However, despite JEV mainly being transmitted by Culicoides and mosquitoes in the natural environment, [17,18,44], we did not detect JEV in the midges collected in Xishuangbanna, Puer, and Dehong. Instead, we found GI-b genotype of JEV in the midges collected from the low incidence area of Zhenkang County, Lincang City [43]. This strain was closely related to the virus strain from Dehong, Yunnan in 2010. This suggests that local midges play a certain role in the transmission of JEV, requiring enhanced monitoring and control of midges and their infection with JEV in the future. In our study, we found that AKV was detected only in Lushui among the 15 locations we sampled. However, Xie [45] detected AKV antibodies in bovine sera collected from 15 different locations, except for Ruili, along the Yunnan border in 2017–2018. This discrepancy might be attributed to differences in host populations, sampling methods, detection method sensitivities, as well as sample quantity and representativeness. Therefore, further exploration of more vector insect midge samples is needed to understand their carriage of AKV and genetic variation. Long-term monitoring of bovine and ovine infections is also required to reduce the harm of AKV to livestock husbandry. In our study, we also obtained the gene sequences of three common vector-borne viruses: EHDV, TIBOV, and BTV in the genus *Orbivirus*. Among them, EHDV was detected only in the border areas adjacent to Myanmar, while TIBOV and BTV were detected in areas bordering Myanmar, Vietnam, and Laos. These viruses are widely present in Yunnan’s border regions and may exhibit regional variations. Therefore, we need to strengthen the monitoring of midge distribution and conduct in-depth research on the carried vector-borne viruses to reduce their impact on livestock husbandry and public health. These findings provide valuable information to better understand the transmission and impact of these viruses, serving as an important basis for future prevention and control strategies. 

The border areas of Yunnan province have a complex and diverse climate with high temperatures throughout the year. The hot and humid summer is conducive to the breeding of various vector insects. Moreover, frequent trade with Southeast Asian countries creates conditions for the introduction and export of vector-borne viruses. Previous surveys have found a wide range of vector-borne viruses in Yunnan’s border areas, such as JEV, BAV, AKV, Dengue virus (DENV), and Tick-borne encephalitis virus (TBEV). These viruses have multiple transmission vectors and hosts, including mosquitoes, bats, midges, ticks, and birds [46,47,48,49,50].

In this study, four consecutive samples were collected in Lushui County (2017–2020), and the most diverse vector-borne viruses were obtained. Four vector-borne viruses were detected: BAV, EHDV, BTV, and AKV. Prior to this, there was no systematic literature on Lushui midges and their associated vector-borne viruses. This study systematically investigated the vector-borne viruses carried by Lushui midges, and it was also the first in-depth study of midges and their carried viruses in Yunnan’s border areas. Through deep sequencing, the distribution of midges in some border areas of Yunnan province was initially understood, and it was found that neighboring countries’ midges had entered China. Neighboring country midges may enter China and spread viruses, causing disease outbreaks. Therefore, in addition to strengthening midge surveillance, it is necessary to conduct in-depth research on the viruses carried by midges to prevent disease transmission and ensure public health security.

This study has several limitations. Firstly, we conducted sequencing by pooling midges from different sampling locations, which was only pathogen screening and did not investigate midge host animals, making it difficult to fully reveal the epidemiological characteristics of the viruses. Secondly, some potentially important parameter data, such as daily weather, temperature, humidity at sampling locations, and surrounding human activities, were not collected, limiting our ability to uncover the influence of various factors on viral composition. Lastly, in this study, we only focused on the genetic characteristics of vector-borne viruses. The potential pathogenicity of other identified viruses in animals, humans, and other mammals remains unknown. According to the current pathogen screening results, future research can focus on host animals, such as mammals and birds, conducting serological surveys to understand their infection status and epidemiological characteristics of vector-borne viruses in the area. This will help reveal the interactions between viruses and hosts, transmission routes, and potential risks of diseases. Besides host animals, future studies can also focus on local vector insects, such as mosquitoes and midges, to explore their role in the virus transmission chain. Investigating and monitoring the types, density, and seasonal distribution of vector insects will provide a better understanding of the dynamics and transmission risk of vector-borne viruses.

Based on the current research findings, it is recommended that we establish a comprehensive disease monitoring and early warning system, monitoring the local vector-borne virus infection and epidemic situation in a timely manner. This includes establishing a robust network for sample collection and testing, enhancing the collection and analysis of epidemic data, and improving the disease diagnosis capacity of medical institutions and health departments. These efforts will enable earlier detection and response to potential epidemic outbreaks, ensuring public health security.

## Data Availability

The sequence reads after QC generated from the 37 libraries generated in this study have been deposited in the NCBI Sequence Read Archive (SRA) database under the BioProject accession numbers PRJNA1044033. The genome sequences of all the viruses generated in this study have also been deposited in GenBank and assigned accession numbers OR872519, OR872520, OR872521, OR872522, OR872523, OR872524, OR872525, OR872526, OR872527, OR872528, OR872529, OR872530, OR872531, OR872532, OR995758, and OR995759.
